# Supplementary material for: Linkage mapping and QTL analysis of flowering time using ddRAD sequencing with genotype error correction in Brassica napus
Source: BMC Plant Biol. 2020 Dec 7;20:546. doi: 10.1186/s12870-020-02756-y (PMC7720618; doi:10.1186/s12870-020-02756-y)
Supplement: Supplementary file 2 — Additional file 2: Fig. S1. Segregation distorted loci across all chromosomes. The significance threshold (p > 0.01) is shown as a red dotted line. Fig. S2. Comparison of linkage group sizes in an uncorrected genetic map and a corrected genetic map. Corrected linkage groups are aligned centrally to uncorrected groups. Fig. S3. Physical (x-axis) and genetic (y-axis) marker positions on all chromosomes in Mb and cM respectively. Spearmans’s rank correlation test result shown in the top left corner of each plot. Fig. S4. Effect plot for the budding time (left) and flowering time (right) QTL on C2 at positions 4,673,904 and 4,655,461 respectively. The ‘AA’ genotype is BnSOSR and the ‘BB’ genotype is BnWOSR. [file 12870_2020_2756_MOESM2_ESM.pdf]

Supplementary figures

Linkage mapping and QTL analysis of flowering time using ddRAD sequencing with genotype error correction in *Brassica napus*

Armin Scheben[1,2], Anita Severn-Ellis[1], Dhwani Patel[1], Aneeta Pradhan[1], Stephen Rae[3], Jacqueline Batley[1], David Edwards[1*]

[1]School of Biological Sciences and Institute of Agriculture, The University of Western Australia, Perth, WA, Australia

[2] Simons Center for Quantitative Biology, Cold Spring Harbor Laboratory, Cold Spring Harbor, NY, 11724, United States of America

[3]BASF Agricultural Solutions Belgium NV, BASF Innovation Center Gent, Technologiepark-Zwijnaarde 101, 9052 Gent

Supplementary figures

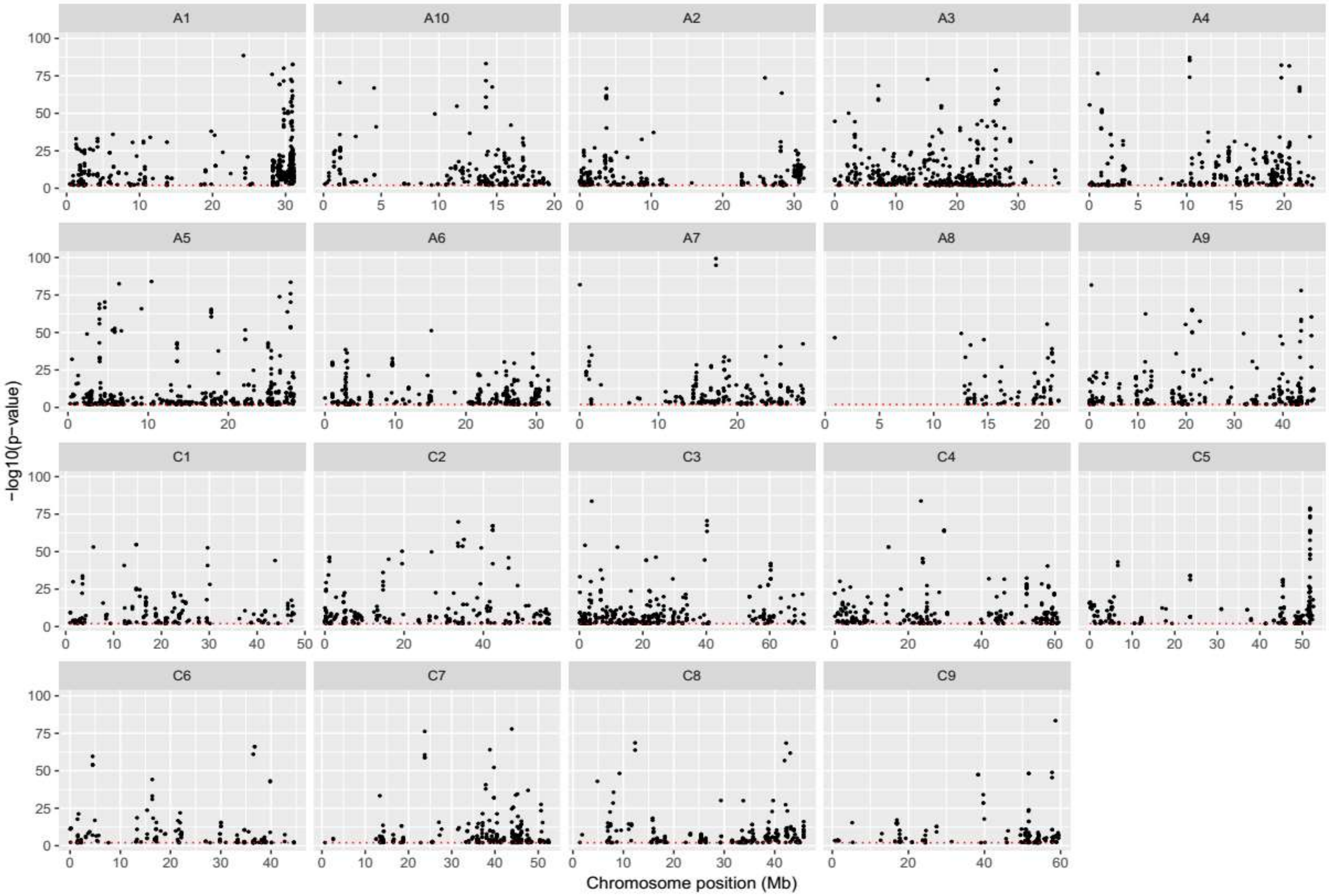

Figure S1. Segregation distorted loci across all chromosomes. The significance threshold (p <0.01) is shown as a red dotted line.

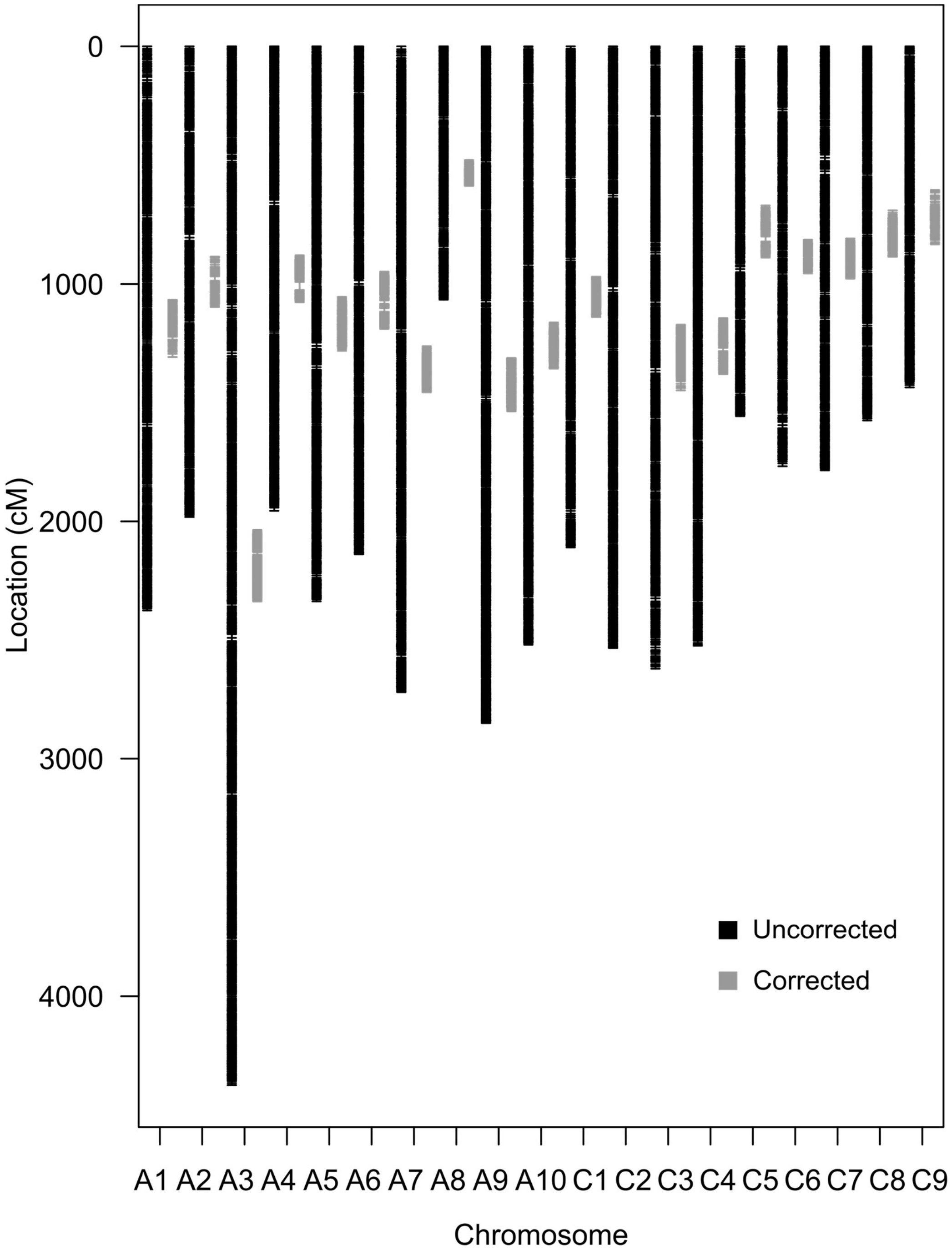

Figure S2 Comparison of linkage group sizes in an uncorrected genetic map and a corrected genetic map. Corrected linkage groups are aligned centrally to uncorrected groups.

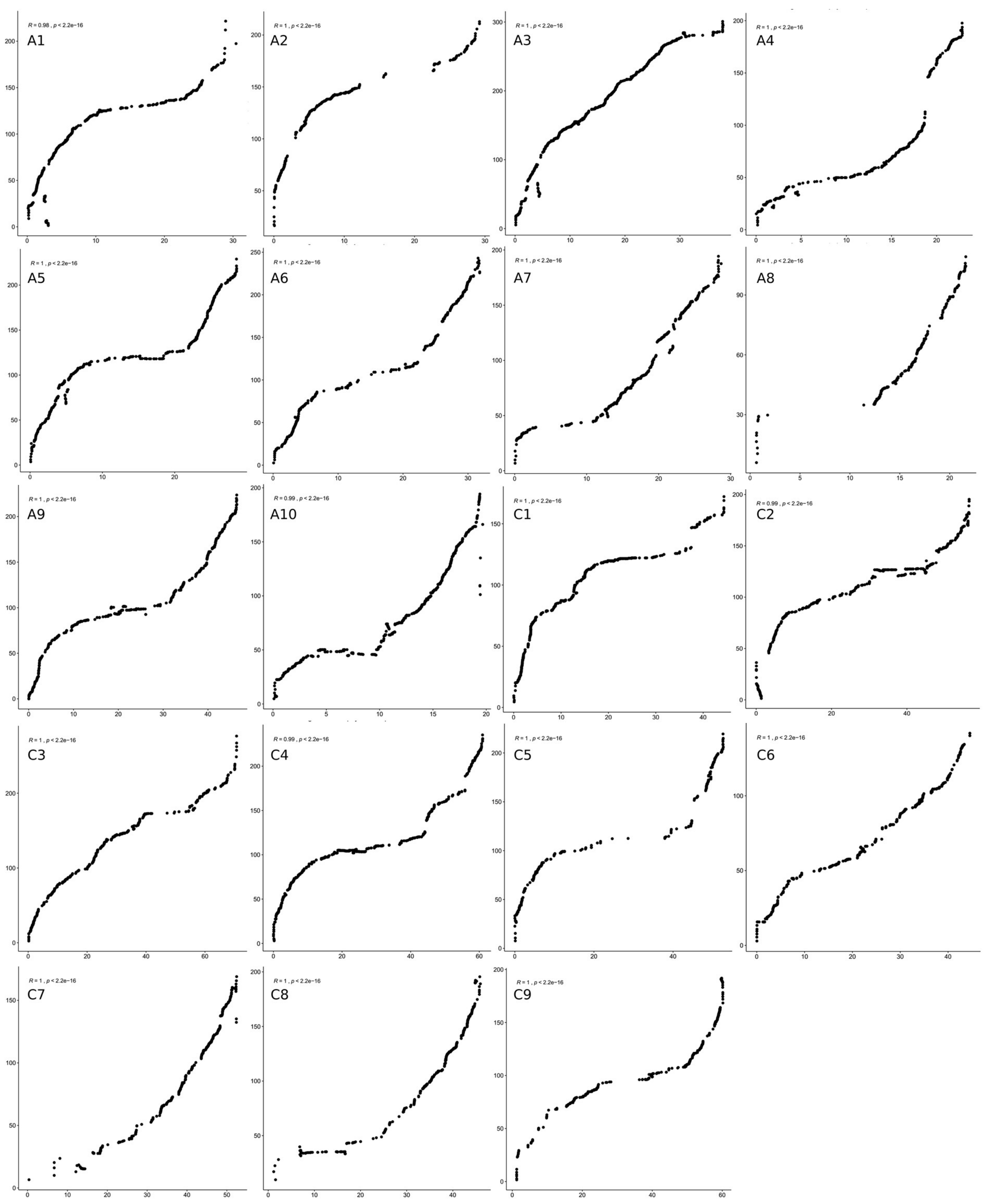

Figure S3 Physical (x-axis) and genetic (y-axis) marker positions on all chromosomes in Mb and cM respectively. Spearmans's rank correlation test result shown in the top left corner of each plot.

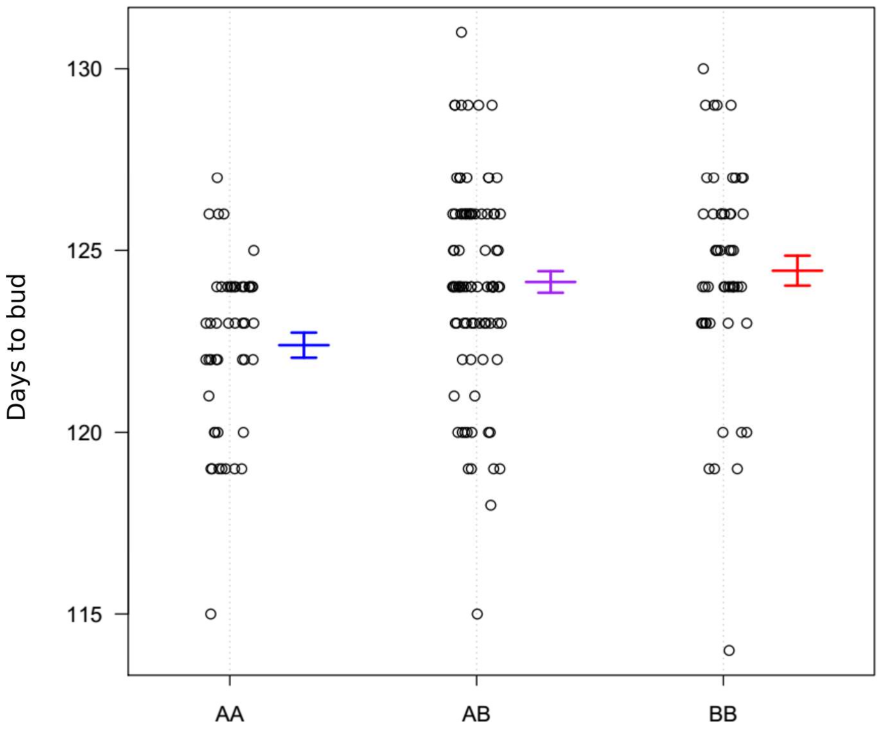

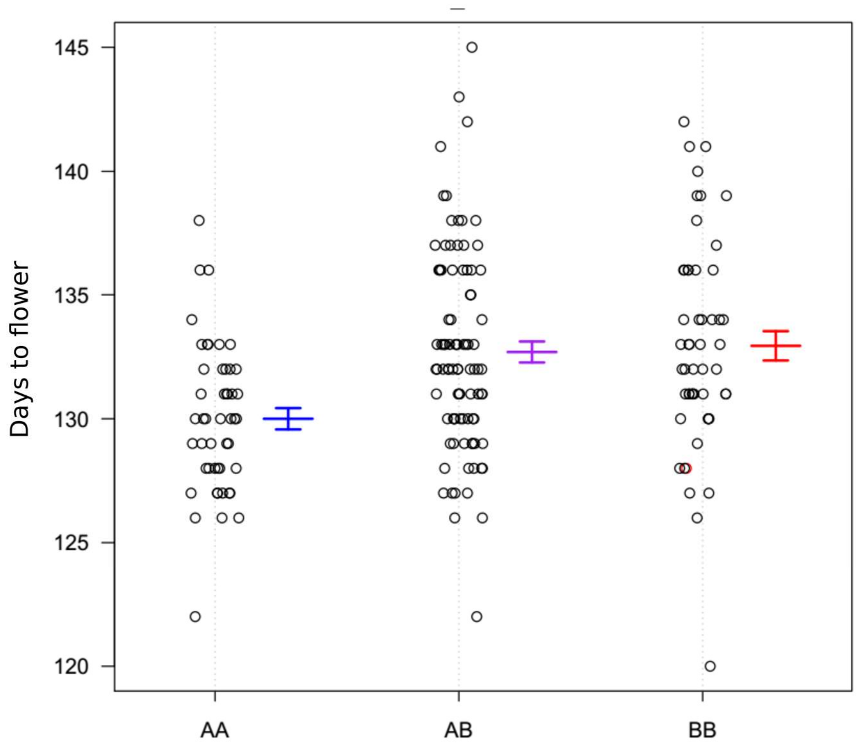

Figure S4 Effect plot for the budding time (left) and flowering time (right) QTL on C2 at positions 4,673,904 and 4,655,461 respectively. The 'AA' genotype is BnSOSR and the 'BB' genotype is BnWOSR.
